# Intra-Dental Abscess. Use of a Vent in the Stopping of a Decayed Tooth

**Published:** 1843-06

**Authors:** Edward Saunders

**Affiliations:** Surgeon Dentist. London Lancet


### ARTICLE VII.

### *Intra-dental Abscess. Use of a Vent in the Stopping of a Decayed Tooth.*

*To the Editor—Sir:*

Cases of suppuration of the lining membrane of the internal cavity of a tooth, and in which the periosteum is not implicated, are, so far as my experience enables me to judge, not of frequent occurrence. More usually inflammation of that delicate and highly organized membrane rapidly extends to that investing the root, and then the whole tooth becomes slightly projected and exquisitely painful; nor is it, probably, ever wholly unaccompanied by inflammatory action in the latter structure. It does, however, occasionally happen that on the sudden exposure of the internal cavity by the breaking down of the thin layer of softened and carious bone which protected it, and more especially if pressure be applied by the injudicious introduction of some substance by way of stopping, that suppuration very speedily ensues. If, under these circumstances, the substance which has been introduced to seal up the cavity be now removed, so as to allow the free discharge of the pus, the inflammatory action appears to subside, and the lining membrane becomes the seat of permanent abscess. This, though it is not susceptible of a radical cure, being unaccompanied by any considerable tenderness, does not interfere with the usefulness of the tooth, the secretion of pus being very minute, scarcely amounting to a spot in the twenty-four hours, and any inconvenience arising from its communicating an unpleasant odor or taste may be easily obviated by introducing a pledget of dry soft lint.

The case, however, is far different if, under these circumstances, we attempt to seal up the cavity by the introduction of a permanent stopping, and an entirely new train of symptoms presents itself. After a short time, varying according to the rate of secretion and the extent of suppurating surface, the pressure of accumulated and pent up matter produces intense pain and inflammation in the remaining healthy portion of the membrane, and, eventually, producing absorption of the parietes of the minute canal at the extremity of the root, escapes into the alveolus, and is finally discharged either at the mouth of the socket and edge of the gum, or through an opening which it effects opposite the end of the root. In this aggravated state of things, the case has, of course, become one of common alveolar abscess, of which it is not my intention here to say anything, my few remarks simply having reference to that form of tooth-disease in which a small portion of the lining membrane of the internal cavity (*cavitas pulpæ*) may be the seat of suppuration, without involving total loss of vitality of the tooth, or inflammation, and consequent tenderness of the periosteum and root.

These remarks were suggested by the following case, which occurred during the past month, and which as it shows the possibility of preserving a tooth from further decay which has been the seat of simple internal abscess, may possibly be regarded with interest by some of your readers.

A gentleman from Cambridge, applied to me to have the operation of stopping performed upon the first molar of the lower jaw, left side. The cavity, which was situated in the central portion, was large, but the parietes of the crown were hard and strong, and capable of sustaining the pressure necessary for the introduction of such a mass of stopping as would be required. The tooth was not then, nor had it been for some considerable time past, the source of pain or annoyance, except as affording a lodgment for the debris of food and the putrescent juices of the mouth. To obviate this in some degree the patient had been in the habit of filling the cavity with cotton. This, however, was not only troublesome, but inefficient, from its partially absorbing and retaining the matter it was intended to keep out of the cavity. On removing the cotton which was then in the tooth I discovered, on the under surface of it, a minute discoloured spot, and on examining the floor of the cavity, an almost microscopic foramen might be traced communicating with the central cavity (cavitas pulpæ). On further inquiry it appeared that about twelve months since this tooth had been filled by a country practitioner, and although up to that period it had never been more than occasionally uneasy, the most intense agony supervened after the lapse of about twelve or fourteen hours, with throbbing or "jumping" headache, and increasing febrile excitement, until the stopping, to which the symptoms were so distinctly referred, was removed. The inflammation and tenderness now quickly subsided, but from that period a minute quantity of pus had been observed upon the under surface of the cotton. Being in possession of these particulars, I somewhat reluctantly complied with the wish of my patient, feeling pretty certain that a repetition of the operation, though at this distance of time, would only reproduce the old train of symptoms. However, as he was unwilling to part with the tooth, and the attention it required in its present state being irksome, I resolved at once to fill up the cavity, and yet to allow a vent for the minute quantity of pus that still continued to be secreted. Accordingly, having "*domed* up" a piece of tin-foil, folded six times upon itself, and placed it like an inverted cup over the opening which allowed the escape of the pus, by which I should avoid pressing upon the internal membrane, I proceeded to complete the stopping in the usual way. This was done without the slightest pain being produced, either during or after the operation, and I now purposed to provide for the discharge of matter by drilling a fine canal through the mass of stopping, taking an oblique direction forwards. Of the necessity of this precaution I could not very readily convince my patient, who requested that it might at least be deferred until some uneasiness should seem to demand it. By the next morning, however, the old symptoms having in some degree begun to present themselves, he was very glad to resort to it. On penetrating the little cup shaped support in the centre, I withdrew my drill and found it tinged with matter, and the relief was instantaneous and complete.

It may be said that much has not been accomplished, no cure has been effected. I answer none was attempted, because I believe it to be impossible (at least in the present state of dental surgery) completely to arrest the suppurative process when once established under such circumstances and in such situation. What has been accomplished is this: a tooth which in any other manner would seem to contra-indicate the operation, has been stopped, and thus not only prevented from affording a lodgment for putrescent particles of food, &c. and protected from their decomposing action upon itself, but restored to its perfect functions as a tooth. The little canal constitutes, in fact, a silver tube, which prevents the pus, in its escape, from coming in contact with the dentine or tooth-bone. To guard against the possibility of its becoming filled up, I was careful to make it extremely small, and to give it an oblique direction forwards, selecting of course the lowest portion of the cavity, and these precautions appear to have been so far successful, no return of the symptoms having been observed. In the case of an upper tooth this care would be unnecessary, and the canal might be drilled perpendicularly, any foreign matter being prevented by its own gravity, from entering the cavity.

I could instance several cases which have come under my own observation in which the operation of stopping has been otherwise well and efficiently performed, but for the want of some such precaution, the tooth, after giving rise to the most distressing agony, has been sacrificed. I will allude to only one, the preparation of which is in the museum of St. Thomas's Hospital. It is somewhat remarkable for the fortitude with which the pain was endured, under the strong hope and conviction that it would subside, the tooth being undoubtedly well stopped. A large cavity, which was discovered in the posterior part of the second superior bicuspis, was filled with gold; a few days after a dull throbbing pain was felt, which became more and more distinctly referrible to this tooth. The stopping being perfect, however, and the tooth not having been previously tender or painful, it was patiently endured for upwards of a week, when headache and fever having supervened, and little or no relief following the application of leeches or fomentation, the tooth was removed. On examining it, I found a large cavity produced in the substance of the root by the pressure of matter thrown out from the internal membrane and pulp. The external wall of this cavity was extremely thin, and in one part there was an actual perforation. It was evident that either the pressure of the stopping upon some part of the lining membrane had set up a suppurative process, or that having previously occurred, the matter was in the same manner confined. In either case the tooth might doubtless have been preserved by the timely adoption of some such plan as I have here recommended. With many apologies for occupying so much of your valuable space on a subject which may probably interest only a portion of your readers. I am, sir, your obedient servant, EDWARD SAUNDERS.

*London Lancet, December* 3, 1842.]

---

*Vent in the Tooth with Intra-Dental Abscess. To the Editor*—SIR: Mr. Saunders, in his treatment of intra dental abscess, appears to have overlooked a cause that must prevent his plan of drilling the inserted "stopping" in teeth, from being likely to meet with continued success. With whatever degree of firmness tin-foil may be inserted into a carious tooth, in cases in which the hole has been drilled, substances of a hard nature will, in mastication, from the malleability of the metal, close the vent in a short time, and this defect will be increased by the hole being drilled in an oblique direction, although were it otherwise, the hole would, as Mr. S. says, be closed by a portion of food remaining in it after eating. And this objection would be the same were the stopping composed of gold instead of tin. I think a better method of giving vent for the escape of the pus is by drilling a hole from the neck of the tooth to the floor of its cavity; and, after the stopping has been inserted, probing the canal to see that it remains clear. This appears to me the only means that can *continue* successful. The hole will require to be drilled with such precision and care (in order to insure its opening exactly on the floor of the cavity,) as must be employed by every member who would successfully practise the profession. I am aware that there may be an objection started to this method, viz. that the ivory or dentine of the tooth, being exposed, is liable to become affected by caries. The canal, however, will be made in a part of the bone *which is then in a healthy state,* consequently a long time will elapse before it becomes diseased. I treated a case in this manner, about two years since, which I had an opportunity of seeing only three weeks back, and I am happy to say it still continued successful. I am, Sir, your obedient servant,*

Samuel Grimes, *Surgeon Dentist.*

*London Lancet,* Dec. 17, 1842.

* The practice recommended in the foregoing communications, quoted from the "London Lancet," is not new in the United States. It was described to one of the editors of this Journal, thirteen years ago, by Dr. L. S. Parmly, and he has adopted it in many instances, but, instead of giving vent through the substance of the filling, he has been in the habit of perforating the neck of the tooth, in the manner as recommended by Mr. Grimes, and afterwards inserting a hollow gold screw for the purpose of protecting the parieties of the opening against the corrosive agents to which they are necessarily exposed. He also has two teeth which were sent to him, some six or eight months since, by Mr. W. H. Elliot, dentist, of Plattsburg, N. Y. with tubed fillings in them.

The practice, however, is unscientific, and should only be adopted as a dernier resort. We believe it would be better, in all cases of sanious discharges from the roots of teeth, to extract the diseased organs. H.

